# Double Aortic Arch, Double SVC, and Coronary Artery Anomaly in a 38-Year-Old Woman

**DOI:** 10.1016/j.jaccas.2025.104937

**Published:** 2025-08-13

**Authors:** Jinye Liu, Thomas Davis, Senad Fazlioglu, Sami Alkoutami, Diana Puicea, Mohamed Elganainy, Abdelrahman Abdelwahed, Khaled Elhusseiny, Cindy Trinh, Sunil J. Desai

**Affiliations:** a Department of Internal Medicine, ECU Health, Greenville, North Carolina, USA; b Department of Internal Medicine, Carilion Clinic, Greenville, North Carolina, USA; c Department of Internal Medicine, WakeMed, Greenville, North Carolina, USA; d Cary Cardiology, PA, Dunn, North Carolina, USA

**Keywords:** congenital heart disease, congenital vascular abnormality, double aortic arch, double superior vena cava, left superior vena cava, single coronary artery

## Abstract

**Background:**

Double aortic arches are rare congenital heart defects, representing <1% of congenital heart defects; most double aortic arches form a vascular ring wrapping around the trachea and esophagus. This finding typically presents in childhood with symptoms like dyspnea, stridor, and recurrent respiratory infections. Adults less commonly present with this condition; however, when they do, respiratory and gastrointestinal symptoms may be present.

**Case Summary:**

We present a case of a 38-year-old woman who was evaluated for dyspnea and chest pain in the emergency department, and incidentally found to have a right-dominant double aortic arch, persistent left superior vena cava, and coronary artery anomaly.

**Discussion:**

The presence of these cardiac vascular anomalies is an exceptionally rare combination not previously reported together. Approaches to initial work-up and future follow-up is addressed.

**Take-Home Message:**

Awareness of these anomalies is critical because they can have major implications regarding future interventions, like catheter-based procedures and surgical planning.

## Graphical Abstract

**Figure:**
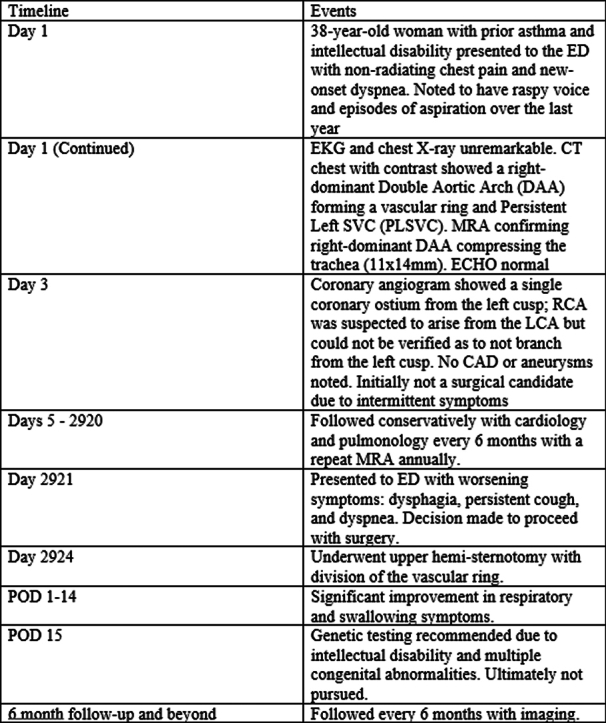

Vascular rings are congenital anomalies of the aortic arch which often compress the trachea and esophagus.^1^ A double aortic arch (DAA) is the most common cause, at an estimated 30%.^2^ A DAA is caused by failure of the right fourth aortic arch to regress, subsequently leading to 2 aortic arches. This anomaly represents <1% of congenital heart defects.^2^

Vascular rings are typically diagnosed in childhood, with a median age of 11 years.^2^ In both younger and adult patients, the vascular ring, usually around the fourth thoracic vertebral level, leads to compressive symptoms such as stridor, recurrent respiratory infections, dyspnea, and cough. Computed tomography (CT) is the preferred imaging for definitive diagnosis and establishing anatomy.^1^ Treatment is predominately surgical and indicated for patients with tracheal or esophageal compression symptoms.^1^

Normal venous anatomy consists of a right-sided superior vena cava (SVC), which is formed from the meeting of the left and right brachiocephalic veins. In some cases, the left brachiocephalic vein continues to form a left-sided SVC.^3^ Persistent SVC occurs when the left anterior cardinal and part of the left common cardinal veins fail to regress during embryogenesis.^3^ In up to 90% of cases, a right SVC is also present, resulting in a double SVC. Although often asymptomatic, this anomaly may occasionally cause arrhythmias and cyanosis.^4^

Single coronary artery is a rare anomaly, reportedly present in 0.024% to 0.066% of the population.^5^ This variation occurs when a single coronary artery arises directly from the aorta in the sinus of Valsalva (aortic sinus), thus that artery is responsible for providing the blood flow for the entire myocardium.^5^

## History of presentation

In March 2015, a 38-year-old woman was seen in the emergency department for nonradiating chest pain without resolution with rest, and dyspnea. Physical examination was unremarkable other than raspy speech.

## Past medical history

She had a permanent caregiver for intellectual disability from an unspecified source, raspy speech, and was a lifelong nonsmoker with no alcohol or illicit drug use. She had an unofficial diagnosis of asthma (no official pulmonary function tests done) because of poor exercise tolerance as a child. On questioning, she mentioned the dyspnea had been relatively new, ongoing for about a year, and also endorsed several aspiration episodes with fluids, and the occasional feeling of pills lodged in her throat when she took them during this time. Her caregiver also described that she had a hoarseness to her breathing and episodes where the patient would struggle to breathe and gasp for air.

## Differential diagnosis

Differentials included cardiac etiologies such as acute coronary syndromes and heart failure. Gastrointestinal disorders such as dyspepsia, reflux, oropharyngeal dysphagia, and lung disorders such as asthma were also considered.

## Investigations

**Figure 1:**
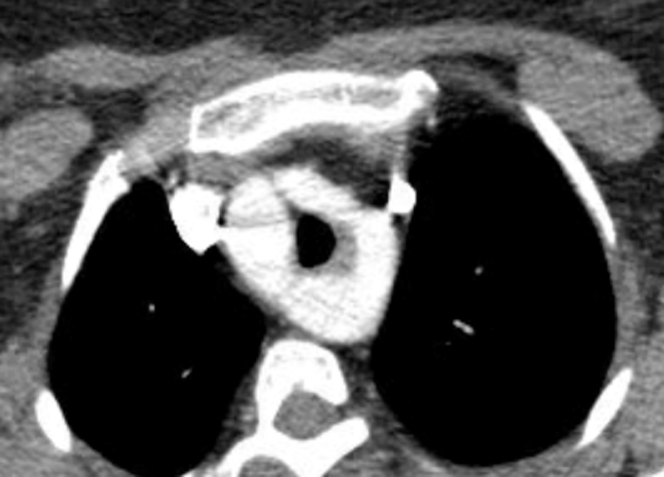
Computed Tomography–Enhanced Image of Vascular Ring Around Trachea With Persistent Left Superior Vena Cava

**Figure 2:**
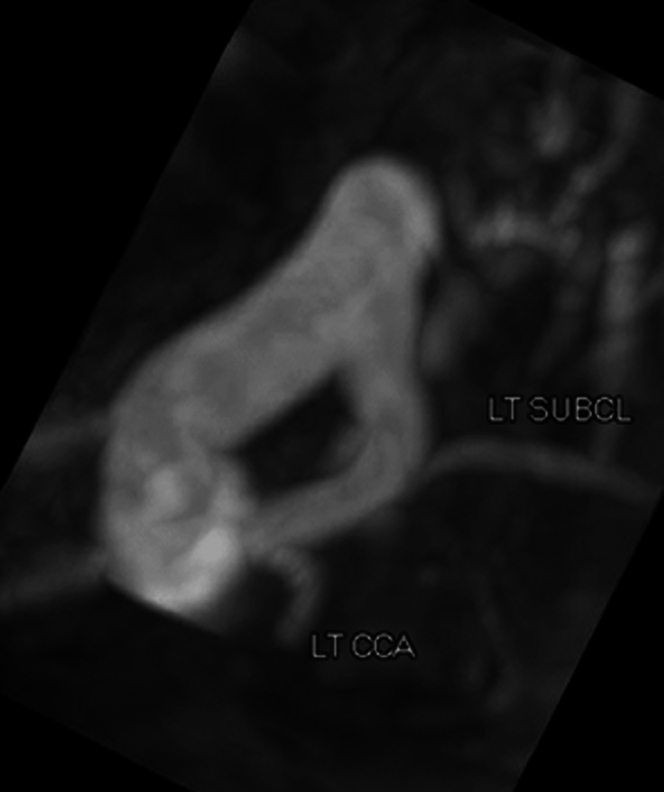
Magnetic Resonance Angiogram View of Double Aortic Arch Forming Vascular Ring From Above LT CCA = left common carotid artery; LT SUBCL = left subclavian artery.

An electrocardiogram and chest radiograph demonstrated no pertinent findings, but a CT obtained to rule out pulmonary embolism revealed an undiagnosed congenital variant DAA and a left persistent SVC (Figure 1). Ancillary testing with an echocardiogram was unremarkable; however, a magnetic resonance angiogram (MRA) revealed a right-dominant DAA (Figure 2). The vascular ring, formed from the DAA, was noted to compress her trachea to a cross-sectional area of 11 × 14 mm at vertebral level T3, possibly explaining her vocal hoarseness and breathing struggles. The left persistent SVC was identified connecting to the normal right-sided SVC through a small brachiocephalic vein and was seemingly an incidental finding.

## Management

**Figure 3:**
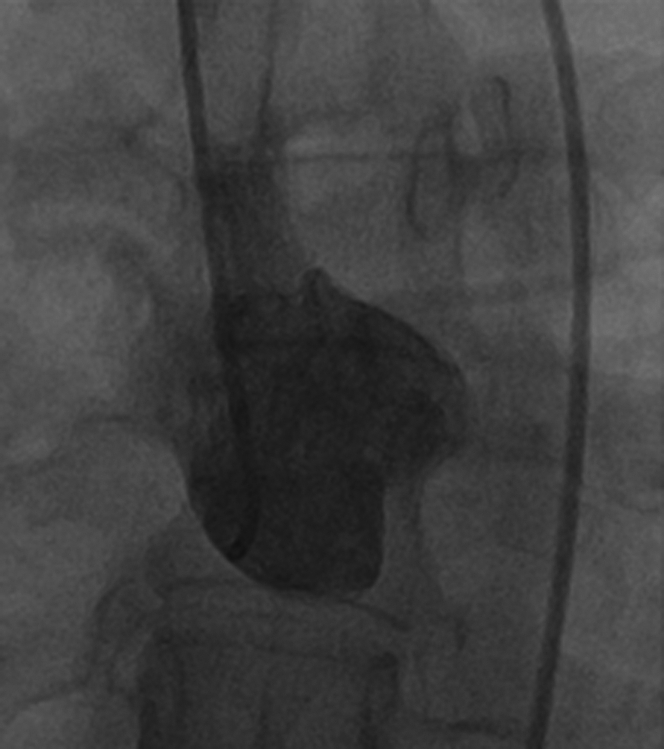
Coronary Angiogram Shows Engagement of Right Cusp of Aortic Valve Demonstrating No Right Coronary Artery

**Figure 4:**
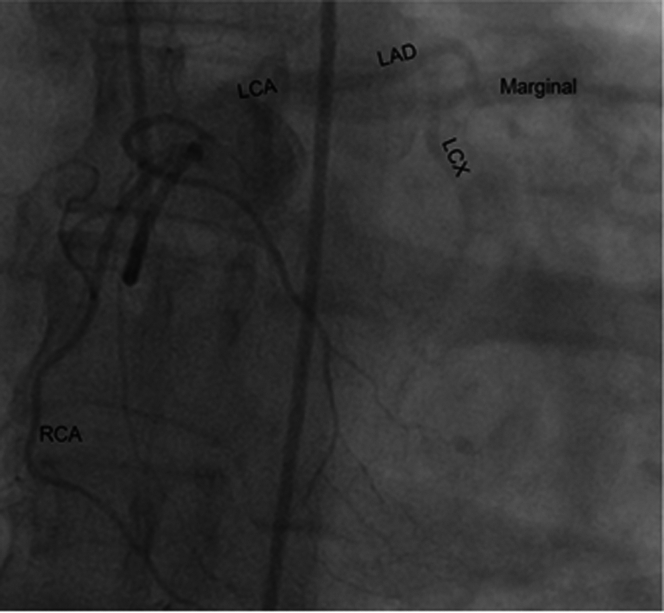
Coronary Angiogram of Left Coronary Ostium on the Left Cusp of the Aortic Valve Demonstrates RCA Branching Off What Appears to be the LCA vs the Left Cusp LAD = left anterior descending; LCA = left coronary artery; LCX = left circumflex artery; RCA = right coronary artery.

She was later seen by cardiothoracic surgery and underwent a full-heart catheterization at a tertiary facility. A single angiogram in the straight anteroposterior and lateral projections in the right sinus of Valsalva demonstrated no right coronary ostium (Figure 3). Instead, there was a single coronary ostium off the left cusp. Engagement revealed engagement of the right coronary artery (RCA) with faint filling in the left coronary artery system; however, on further review, it was highly suggestive that the RCA branched off the left coronary artery rather than the left cusp, but could not be definitively verified. There was no significant stenosis, aneurysms, fistula, or evidence of coronary artery disease (Figure 4). It was thought that the coronary artery anomaly was noncontributory to the patient's symptoms.

## Outcome and follow-up

**Figure 5:**
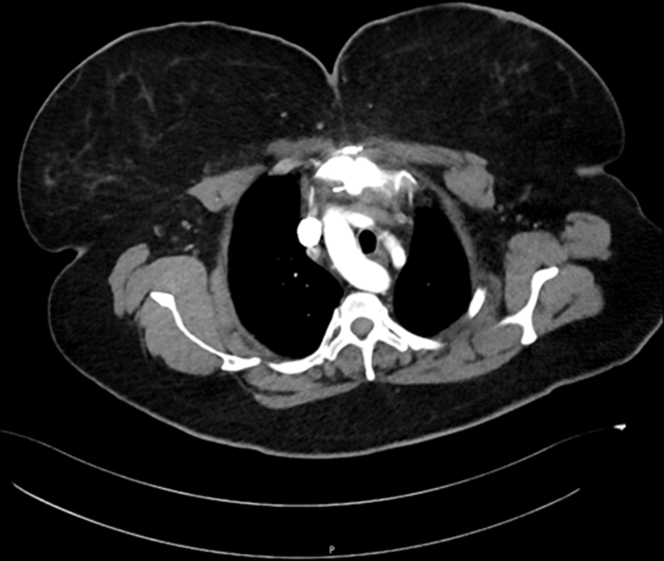
Computed Tomography Scan 6 Months Postoperative Noting Ligation of the Distal Aortic Arch Without Patency

The patient originally was not deemed a surgical candidate because she was without consistent and severe periods of respiratory or gastrointestinal symptoms; therefore, she continued to follow-up with cardiology and pulmonology every 6 months, with a repeat MRA annually. However, during her recent follow-ups, she presented with uncontrollable dysphagia, coughing, and shortness of breath, prompting surgical intervention. She underwent an upper hemisternotomy with division of her vascular ring by cardiothoracic surgery. This process included creating a pericardial well, dissecting the aortic arch out, and placing a vessel loop around the anterior limb of the vascular ring between the left common carotid and left subclavian arteries, with the stumps sewn closed. Postoperatively, the patient had considerable improvement in her symptoms, and is getting repeat imaging every 6 months (Figure 5). The patient was recommended to undergo genetic testing given her concurrent intellectual disability and congenital heart defects; however, this was never followed-up on.

## Discussion/Conclusions

Normally, there are a pair of 6 aortic arches, persistence or involution of these leads to various congenital anomalies. Around the first month of gestation, the right-sided arch typically regresses, leaving a normal left-sided arch. However, in a patient with a DAA, there are 3 possible variants. Most common is a right dominant system, with estimated prevalence of 66% to 80%.^1^^,^^2^ Other variants include a balanced and left dominant system, which occur less frequently at 5% to 10% each.^1^^,^^2^ DAA anomalies usually occur in isolation; however, 12% to 63% of cases can be associated with other cardiac pathology, such as ventricular septal defects, atrial septal defects, coarctation of aorta, and tetralogy of Fallot.^2^

The most common presenting symptom in children with DAA is dyspnea, followed by recurrent respiratory infections, stridor, cough, and wheezing.^2^ Bacterial growth is common because of airway obstruction that causes mucus buildup. Adults were previously thought to have more gastrointestinal symptoms (gastroesophageal reflux disease and dysphasia) than respiratory; however, a recent cohort study suggests that there is no significant difference in respiratory or gastrointestinal symptoms in adults presenting with vascular rings.^2^ It is very likely that our patient had her initial DAA presentation as a child with her asthma; however, no further work-up was done at that time.

Definitive diagnosis is through CT imaging, which establishes anatomy. Echocardiogram, pulmonary function tests, and barium esophagrams are also important ancillary tests. They aim to establish other congenital defects because they are a common phenomenon in patients with DAA. Surgical division of the DAA is the treatment for severely symptomatic patients and is generally well tolerated.

Persistent left SVC is the most common congenital venous anomaly of the thorax, which occurs in 0.5% of the general population and 10% of those with congenital heart disease.^3^ Although mostly asymptomatic, this does pose possible issues during placing central lines or during catheter ablation procedures. This was an incidental finding in our patient.

Furthermore, not only are there extremely few reported cases of 2 of the 3 conditions, there have not been any reported cases of all 3 together to our knowledge. We suspect that the patient likely had some signaling errors occur in embryologic development which led to her diagnoses, such as in abnormal expression of vascular endothelial growth factor which may be contributory toward her vasculature abnormalities, or defects in neural crest cell migrations which may affect development of the aortic and pharyngeal arches. DiGeorge syndrome remains a possibility, and her intellectual developmental delay is possibly also related to these signaling errors; however, these remain unproven because of a lack of genetic testing. Although our patient is fortunate to be asymptomatic from 2 of these anomalies, she was symptomatic from her vascular ring and the compressive effects it has created.

This case highlights the shift in the previous paradigm that adults who present with a vascular ring have gastrointestinal predominant symptoms. Furthermore, our patient demonstrates that while a CT can establish diagnosis, computed tomography angiography or MRA often best demonstrates true vascular anatomy because our patient had several congenital anomalies discovered on this work-up. Furthermore, knowledge of these vascular anomalies in patients is essential in surgical planning and for future procedures like catheter ablations, coronary CTAs, or cardiac catheterizations, because unanticipated anatomy can lead to procedural delays, difficulties, or complications.

**Visual Summary:**
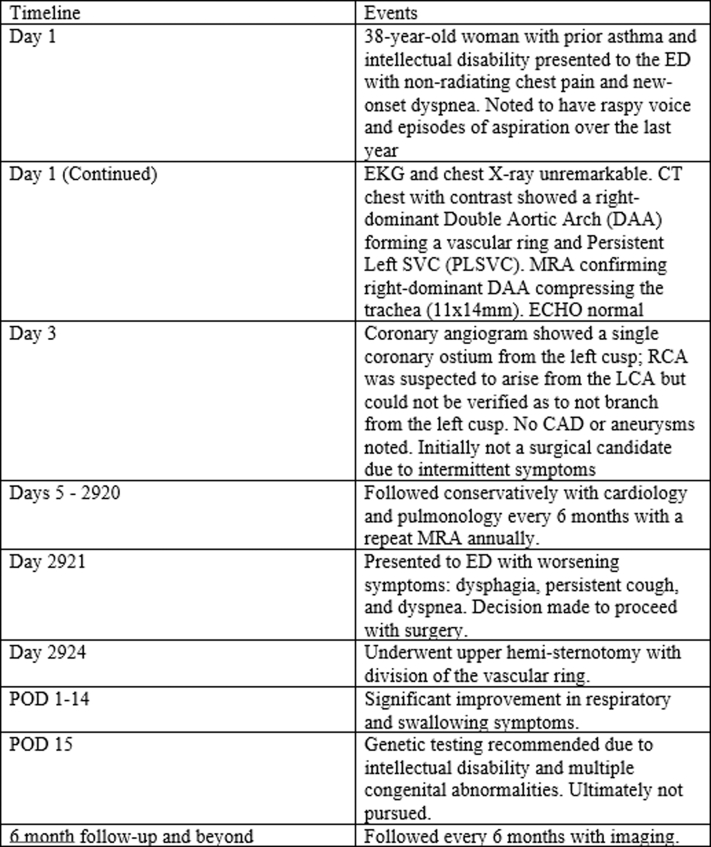
Timeline of Events From Presentation to Postoperative Follow-Up CAD = coronary artery disease; CT = computed tomography; ECHO = echocardiogram; ED = emergency department; EKG = electrocardiogram; LCA = left coronary artery; MRA = magnetic resonance angiography; RCA = right coronary artery.

## Funding Support and Author Disclosures

The authors have reported that they have no relationships relevant to the contents of this paper to disclose.

**Take-Home Messages**

• Identifying and challenging known assumptions regarding symptoms in adults with congenital vascular abnormalities: this case highlights a paradigm shift in understanding the presentation of vascular rings in adults, and thus challenges the notion that GI symptoms are what predominate and recommends a balanced approach when assessing both GI and respiratory symptoms in these adults with congenital vascular abnormalities.
• Comprehensive imaging as an essential for exact diagnosis and surgical planning: using advanced imaging techniques like MRA/computed tomography angiography for accurately diagnosing patients with complex vascular anatomy, especially those associated with DAA and other abnormalities, helps optimize future patient outcomes by establishing possibly new diagnoses and also helping to stage planning for surgical interventions and other procedures.
